# Neurolymphatic clearance in neurodegenerative disease: Emerging mechanisms and potential translational strategies

**DOI:** 10.1016/j.jpra.2025.12.013

**Published:** 2025-12-17

**Authors:** Adriano Fabi, André S. Alves, Albert Neutzner, Stephan Frank, Ana Lariu, Laurent Muller, Tarek Ismail, Dimitrios G. Gkotsoulias, Raphael Guzman, Dirk J. Schaefer, Nir Grossman, Marc Aurel Busche, Elisabeth A. Kappos

**Affiliations:** a Department of Plastic, Reconstructive, Aesthetic and Hand Surgery, University Hospital of Basel, Spitalstrasse 21, 4031 Basel, Switzerland; b Faculty of Medicine, University of Basel, Klingelbergstrasse 61, 4031 Basel, Switzerland; c Department of Surgery, University Hospital of Geneva, Rue Gabrielle-Perret-Gentile 4, 1205 Geneva, Switzerland; d Department of Biomedicine, University of Basel, Hebelstrasse 20, 4031 Basel, Switzerland; e Institute of Pathology, University Hospital of Basel, Schönbeinstrasse 40, 4031 Basel, Switzerland; f Department of Otorhinolaryngology, Head and Neck Surgery, University Hospital of Basel, Petersgraben 4, 4031 Basel, Switzerland; g Research Center for Clinical Neuroimmunology and Neuroscience, University Hospital of Basel, Petersgraben 4, 4031 Basel, Switzerland; h Translational Imaging in Neurology Basel, Department of Biomedical Engineering, Faculty of Medicine, University of Basel, Hegenheimermattweg 167b, 4123 Allschwil, Switzerland; i Department of Neurology, University Hospital of Basel, Petersgraben 4, 4031 Basel, Switzerland; j Department of Neurosurgery, University Hospital of Basel, Spitalstrasse 21, 4031 Basel, Switzerland; k UK Dementia Research Institute, Imperial College London, 86 Wood Lane, W12 0BZ London, United Kingdom; l UK Dementia Research Institute, University College London, Gower Street, WC1E 6BT London, United Kingdom

**Keywords:** Neurolymphatic system, Glymphatic system, Meningeal lymphatic vessels, Microsurgery, Neurodegenerative Diseases, Neuromodulation

## Abstract

**Introduction:**

Neurolymphatic dysfunction has been linked to cognitive decline and implicated in the pathogenesis of neurodegenerative disorders such as Alzheimer’s Disease (AD) and Parkinson’s Disease (PD). Despite its growing recognition, the potential role of pharmacological or surgical neurolymphatic modulation remains poorly understood.

**Objectives:**

This review summarizes current evidence on the neurolymphatic system’s anatomy, physiology and its involvement in neurodegenerative diseases. It also examines emerging pharmacological and lymphatic reconstructive techniques.

**Methods:**

A comprehensive literature search was conducted in PubMed, yielding 187 studies related to the neurolymphatic system. Studies were screened for the following topics: (1) Anatomy and physiology of the neurolymphatic system, (2) The association between neurolymphatic dysfunction and neurodegenerative diseases, (3) Pharmacological and (4) Microsurgical neurolymphatic modulation.

**Results:**

Current evidence suggests that the neurolymphatic system facilitates drainage of interstitial and cerebrospinal fluid to the deep cervical lymph nodes. Preclinical models suggest that enhancing their clearance may promote the clearance of neurotoxic proteins and potentially improve cognitive function.

**Conclusions:**

Scientific evidence on neurolymphatic modulation in neurodegenerative diseases is scarce. Both pharmacological and microsurgical modulatory techniques remain experimental approaches for neurodegenerative diseases, with a significant potential to improve patients’ quality of life. However, further research is warranted to establish their safety, feasibility, and efficacy. The current knowledge gaps underscore the need for a detailed mapping of the neurolymphatic pathways, preclinical evaluation, and translational interdisciplinary trials.

## Background

Alzheimer’s disease (AD) and Parkinson’s disease (PD) are the most prevalent neurodegenerative disorders.^1^ AD is a progressive neurodegenerative disorder characterized by memory loss, cognitive decline and behavioral changes due to misfolded hyperphosphorylated tau and amyloid-beta (Aβ) aggregates within the brain parenchyma.^2^^,^^3^ Pharmacological therapies targeting Aβ synthesis and aggregation have thus far failed to cure AD.^4^ PD, on the other hand, is characterized by degeneration of dopaminergic neurons in the substantia nigra pars compacta, leading to motor dysfunction, including bradykinesia, resting tremor, rigidity and postural instability.^5^ The defining neuropathological hallmark of PD is the presence of Lewy bodies—intracytoplasmic neuronal inclusions largely composed of misfolded α-Synuclein (α-Syn), but also containing lipid membranes and organelles such as mitochondria.^5^^,^^6^ Both AD and PD significantly affect patients’ and caregivers’ quality of life, and thus pose significant societal and economic burdens.^7^^,^^8^ Over the past decade, increasing evidence has highlighted the presence of functional lymphatic vessels filtering macromolecules, such as Aβ and α-Syn, from the cerebrospinal fluid (CSF) into the deep cervical lymph nodes.^9^^,^^10^ Notably, Chao et al. reported an increased risk of dementia in patients who had undergone bilateral cervical lymph node dissections.^11^ Furthermore, neurolymphatic dysfunction has been linked to cognitive decline in the elderly.^12^ In a mouse model, Wu et al. and Wang et al. both demonstrated impaired brain waste removal after cervical lymph node removal and/or ligation, characterized by accumulation of Tau.^13^^,^^14^ Consequently, directly targeting the lymphatic clearance of Aβ and α-Syn has emerged as a promising new therapeutic strategy to treat or even delay the onset of cognitive decline.^6^^,^^15^, ^16^, ^17^, ^18^

For plastic and reconstructive surgeons, these insights introduce a novel translational field where safe and effective microsurgical principles, such as lymphovenous anastomosis and vascularized lymph node transfer, could extend beyond the management of peripheral lymphedema to the modulation of central lymphatic pathways.^19^^,^^20^ Consequently, the aim of this review is to summarize current evidence of the neurolymphatic system and to explore the potential neuroprotective effects of pharmacological and surgical neurolymphatic modulation. By identifying critical knowledge, this review may guide future investigations into the potential of neurolymphatic modulation.

## Methods

A literature search was performed in PubMed/MEDLINE in March 2025 to identify studies reporting on the anatomy, physiology, or modulation of the neurolymphatic system. The search strategy combined the following key terms: “neurolymphatic system”, “meningeal lymphatic vessels”, “glymphatic system”, “neurodegenerative disease”, “Alzheimer’s disease”, “Parkinson’s disease”, “lymphatic reconstruction”, “lymphatic modulation”, “lymphovenous anastomosis”, and “vascularized lymph node transfer”. Articles of all types and publication years, including case reports, cohort studies, and review articles, were eligible for inclusion, provided they were published in English. Both animal and human studies were included and analyzed separately. Given the exploratory nature of neurolymphatic modulation, findings were narratively summarized to highlight emerging therapeutic implications.

## Anatomy and physiology of the neurolymphatic system

### Extracellular fluids of the central nervous system

**Figure 1:**
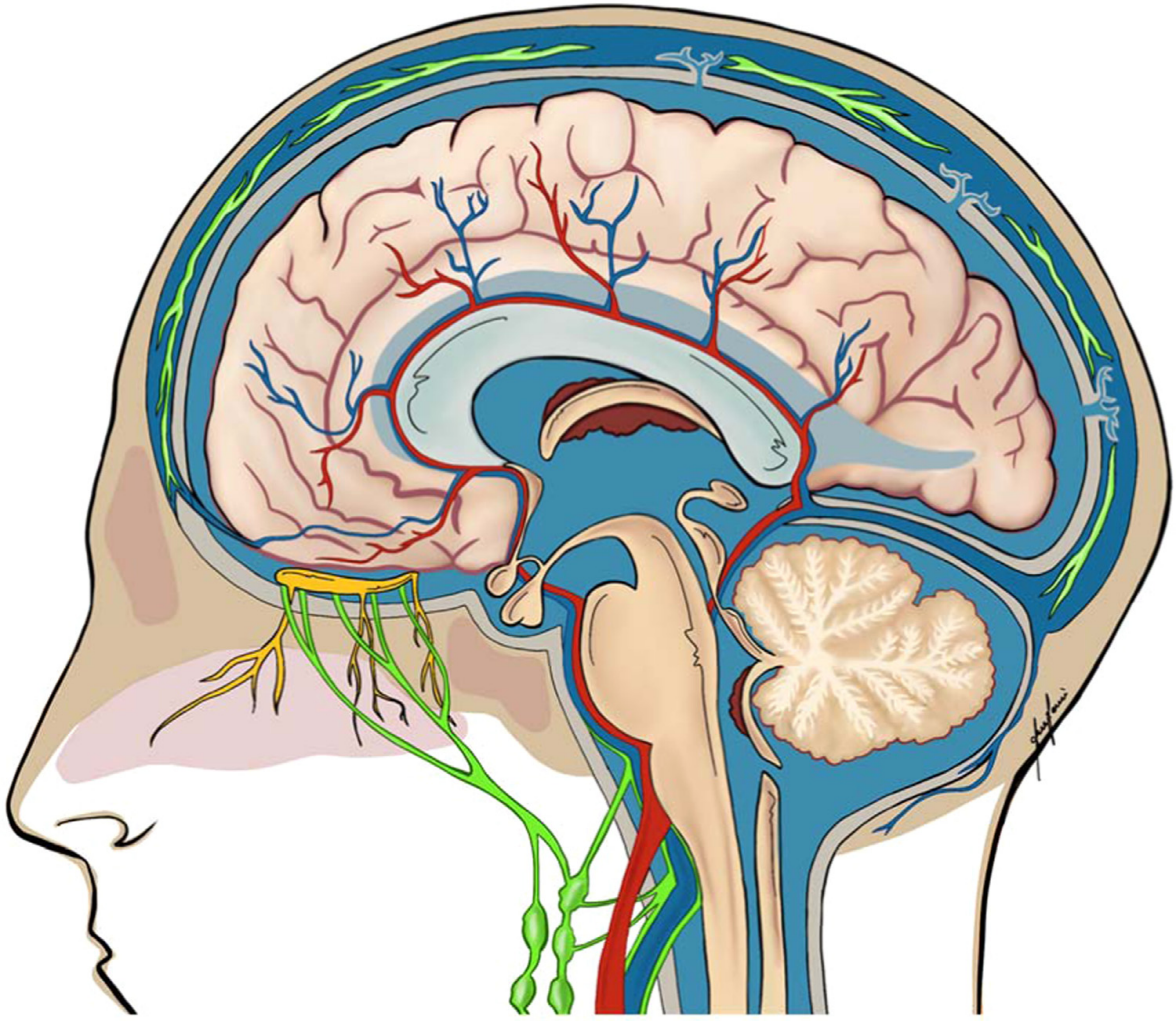
Anatomical pathways of the neurolymphatic system (green). Interstitial fluid is transported along perivascular routes toward the skull base and ultimately into the deep cervical lymph nodes. Cerebrospinal fluid drains via three principal routes: (1) arachnoid villi and granulations into the dural venous sinuses, (2) perineural spaces along the olfactory nerves through the cribriform plate into nasal lymphatics, and (3) meningeal lymphatic vessels located dorsally along the superior sagittal sinus and basally along the petrosquamosal and sigmoid sinuses.

The central nervous system (CNS) contains two extracellular fluid compartments: the cerebrospinal fluid (CSF) located within the cerebral ventricles and subarachnoid spaces, and the interstitial fluid (ISF), dispersed throughout the brain parenchyma.^21^ ISF provides ionic and nutritional balance critical for neuronal function and survival, and removes metabolic waste, while also contributing to the physical protection of neural tissue.^22^^,^^23^ On the other hand, CSF is primarily produced by the choroid plexus and serves as a reservoir for ISF and ensures its compositional stability through continuous exchange.^21^^,^^24^ CSF was historically believed to be solely resorbed by the arachnoid villi, so that the role of extracranial lymphatics in CSF dynamics was largely overlooked until recently, when up to 50 % of CSF was shown to drain via extracranial lymphatic pathways.^15^, ^16^, ^17^^,^^21^ Interestingly, two separate drainage pathways for ISF and CSF have been described recently (Figure 1).^21^^,^^25^^,^^26^

### Perivascular drainage (ISF pathway)

Astrocytic end-feet surround the cerebral vasculature, forming a perivascular space (PVS) approximately 40 µm in diameter, known as the glymphatic system.^27^ The existence and the exact function of the glymphatic model remains highly debated, yet it is believed to directly clear metabolic waste, including Aβ and tau protein, from the ISF.^21^^,^^27^, ^28^, ^29^, ^30^, ^31^ Consequently, the decline in perivascular ISF clearance in the aging brain leads to elevated peptide concentrations and thus predisposes to Aβ plaque accumulation.^12^^,^^27^^,^^32^, ^33^, ^34^ Similarly, dysfunction in perivascular clearance exacerbates the accumulation of neurotoxic proteins and has been associated with early cognitive decline.^12^^,^^34^ Notably, Mawuenyega et al. found late-onset AD to be associated with a 30 % decline in Aβ clearance, and consequently suggested that decreased ISF clearance rates could potentially be used as early predictors for AD.^34^

Interestingly, Aβ plaques have been identified in intracranial arteries and leptomeninges yet remain absent in cervical segments of the carotid artery, as demonstrated in both human and animal studies.^35^^,^^36^ In contrast, Al-Diwani et al. detected various neurodegenerative biomarkers in biopsies of human deep cervical lymph nodes (dcLNs).^10^ This finding implies that the glymphatic network parallels certain lymphatic functions by channeling ISF alongside cerebral vasculature through the skull base into deep cervical lymph nodes.^25^^,^^37^ Similar to AD, accumulating evidence suggests that impaired perivascular and glymphatic function may also contribute to Parkinson’s disease (PD) pathophysiology, with impaired glymphatic clearance of misfolded α-Syn accelerating the progression of Parkinson’s disease.^38^, ^39^, ^40^, ^41^ Notably, glymphatic transport is strongly regulated by the sleep–wake cycle, as non-REM sleep facilitates ISF–CSF exchange.^42^^,^^43^ Interestingly, sleep problems have been associated with glymphatic dysfunction and the accumulation of neurodegenerative metabolites, including Aβ and α-Syn.^44^ Consequently, the perivascular drainage (ISF pathway) has consistently been suggested as a novel potential target for both AD and PD.^21^^,^^32^^,^^38^

### Meningeal lymphatic system (CSF pathway)

The meninges, which envelop the brain and spinal cord, consist of three layers: the pia mater—a monolayer of cells covering the CNS, the arachnoid mater, and the outermost dura mater, which contains blood vessels.^45^ The dura mater contains a large network of meningeal lymphatic vessels (mLVs).^26^^,^^46^, ^47^, ^48^ These dural mLVs drain macromolecules and solutes from the CSF into the deep cervical lymph nodes, with studies estimating up to 50 % of CSF clearance to occur via this lymphatic route.^9^^,^^49^ Three primary hotspots for CSF drainage have been identified:

1. Arachnoid granulations: Arachnoid granulations, or Pacchionian bodies, are macroscopic extensions of the arachnoid membrane that penetrate the dura mater and project into the dural venous sinuses.^50^^,^^51^ Smaller forms of these structures are known as arachnoid villi. The villi and granulations reabsorb CSF into the venous circulation, allowing for intracranial pressure regulation and maintenance of CNS fluid balance.^17^^,^^21^^,^^50^^,^^51^
2. Cribriform plate and nasal lymphatics: The cribriform plate channels fluid from the subarachnoid space into the extracranial lymphatic system.^51^^,^^52^ Experimental tracer studies have demonstrated rapid CSF movement along leptomeningeal arteries toward the cribriform plate, where the CSF follows olfactory nerve bundles through the foramina of the ethmoid bone into the nasopharyngeal lymphatic plexus (NPLP) and ultimately drains into the cervical lymph nodes.^53^^,^^54^
3. Dural lymphatic system: Dorsal meningeal lymphatic vessels (mLVs) are located near the superior sagittal and transverse sinuses.^55^^,^^56^ Interestingly, dorsal mLVs are highly sensitive to vascular endothelial growth factor (VEGF) signaling.^55^ In contrast, basal mLVs are found along the petrosquamosal and sigmoid sinuses and are characterized by larger diameters and the presence of lymphatic valves, making them more effective in clearing CSF and associated macromolecules.^47^^,^^55^ Using T2-FLAIR MRI imaging with CSF contrast injected into the cisterna magna, Ahn et al. observed the basal mLVs to be the main route of CSF drainage in a mouse model.^55^ Similarly, Johnston et al. injected Microfil as CSF tracer into the cisterna magna of various species and observed comparable drainage pathways mostly located at the base of the brain and in the basal cisterns.^53^ Eide et al. acquired T1-w MRI scans repetitively, before and up to 48 h, following intrathecal administration of gadobutrol as CSF tracer, showing evidence of CNS lymphatic drainage to cervical lymph nodes, in vivo.^57^ Absinta et al. utilized T2-FLAIR and T1-w black-blood imaging in combination with gadobutrol to successfully reveal the topography of meningeal lymphatic vessels in human and non-human primates, while recent studies have successfully used non-invasive optimized MR imaging techniques to visualize the dural lymphatic networks and their connections to the cervical lymph nodes, in vivo. Experimental studies demonstrated that ligation of mLVs led to increased amyloid and α-Syn deposition within the meninges and a marked decline in cognitive function.^9^^,^^28^^,^^58^ Similarly, studies have demonstrated that extracranial cervical lymphatic ligation may cause brain lymphatic clearance dysfunction and consequently lead to an accumulation of α-synuclein in the brain parenchyma, culminating in dopaminergic neuronal loss and motor deficits in mice.^41^

### Deep cervical lymph nodes (dcLNs)

The perivascular (ISF) and meningeal lymphatic (CSF) pathways function synergistically and converge at the dcLNs.^9^^,^^10^^,^^28^^,^^35^^,^^36^ In fact, this was also shown in recent MRI studies.^57^^,^^59^ Chao et al. observed that head and neck cancer patients who underwent bilateral cervical lymph node dissection exhibited an increased risk of dementia.^11^ Although their important study suggested a potential neuroprotective role of the dcLNs, it had significant confounders, including radiotherapy in 273 patients, the exclusive inclusion of male patients over 60 years and mixing all types of neck dissection. Nevertheless, both Wu et al. and Wang et al. also observed brain waste accumulation after cervical lymph node removal and/or ligation.^13^^,^^14^ However, given the observational retrospective nature of current research, further studies are needed to establish a causal relationship in humans.

## Pharmacological modulation may improve neurolymphatic outflow

**Figure 2:**
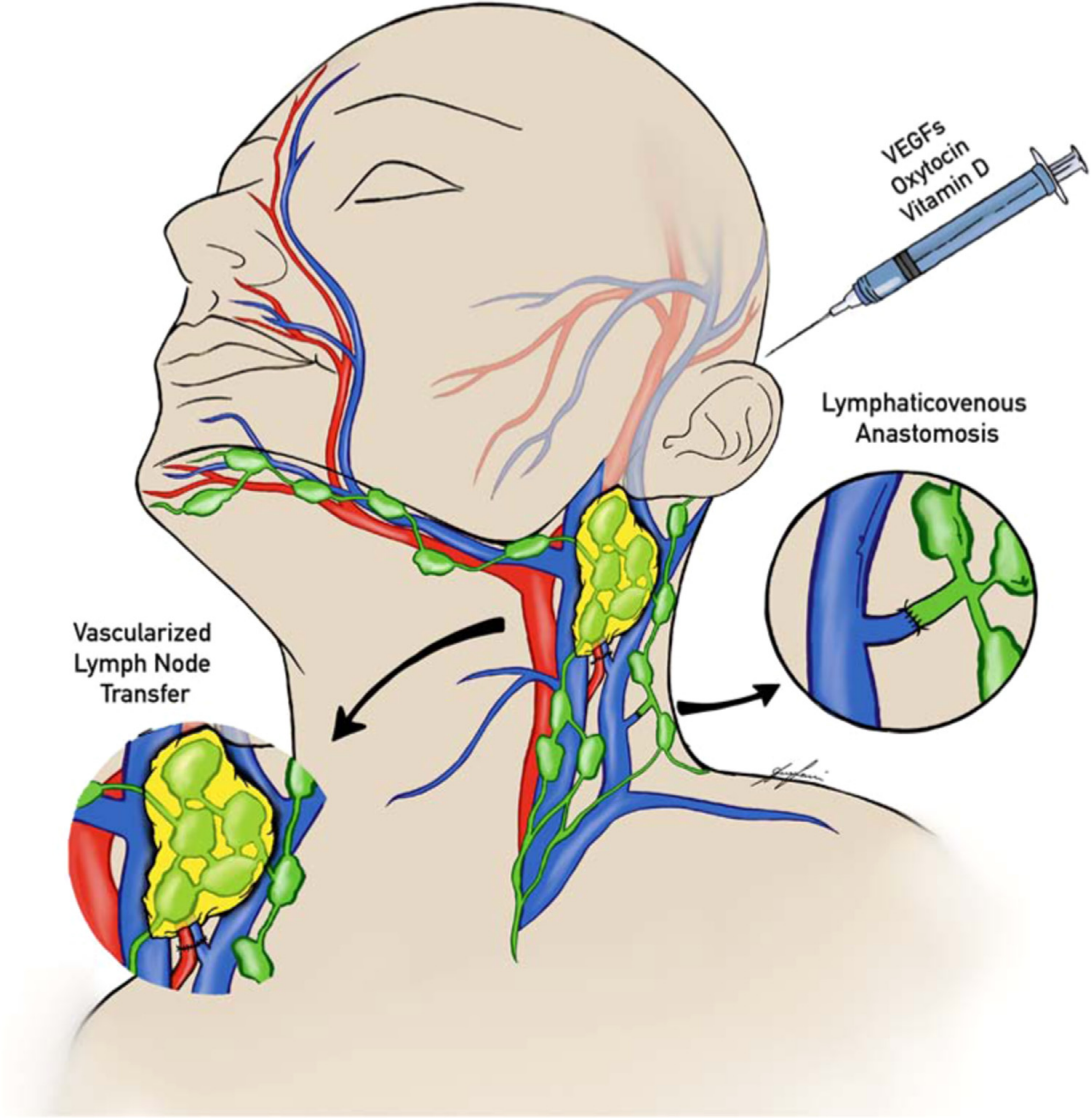
Currently hypothesized translational strategies for neurolymphatic modulation. Lymphovenous anastomosis (LVA) and vascularized lymph node transfer (VLNT) to the neck are believed to enhance lymphatic flow. Pharmacologic agents such as VEGF-C, oxytocin, and vitamin D may further modulate meningeal lymphatic activity.

Lymphatic vessels, including mLVs, exhibit remarkable plasticity and regeneration capacity.^9^^,^^46^^,^^47^^,^^60^ Consequently, pharmacological modulation of meningeal lymphatic drainage may present an opportunity to conservatively improve cognitive performance (Figure 2).^9^^,^^61^ Among the most extensively studied modulators is VEGF-C which binds to VEGFR-3 tyrosine kinase receptors expressed on blood and lymphatic vessels, consequently promoting lymphangiogenesis.^60^^,^^62^ Experimental VEGF-C administration in mice increased lymphatic drainage and improved macromolecular clearance and cognitive function, suggesting a promising regenerative potential for the neurolymphatic system.^9^^,^^63^ Similarly, the intracerebrospinal administration of an adeno-associated virus expressing VEGF-C increased neurolymphatic drainage to the dcLNs by enhancing lymphatic growth in a mouse stroke model.^60^ Finally, injection of VEGF-C into the cisterna magna also stimulated meningeal lymphangiogenesis, which was associated with decreased neuroinflammation and improved motor functions in cirrhotic rats.^61^ More recent but understudied pharmacological approaches include oxytocin (OT) and vitamin D. Administration of OT into the cisterna magna of AD mice promoted Aβ transfer from the brain parenchyma to the CSF, suggesting potential clearance of Aβ. Additionally, inflammatory cytokines were significantly decreased after OT administration.^28^ In rats with subdural hematoma, treatment with vitamin D promoted hematoma clearance into the dcLNs by improving structure and function of meningeal lymphatic vessels.^64^ However, further evidence is required to substantiate these preliminary findings.

## Microsurgical lymphatic reconstruction potentially improves cognitive function

(Super-)microsurgical lymphatic reconstruction is a well-established treatment designed to improve lymphatic drainage and alleviate lymphedema-associated symptoms in patients across various indications, including lymphedema of the extremities.^20^^,^^65^ Given that neurolymphatic outflow has been observed to correlate with disease onset, progression and severity, there is growing interest in exploring whether (super-)microsurgical lymphatic reconstruction may modulate neurolymphatic clearance and cognitive function.^66^

## Lymphovenous anastomosis

Lymphovenous anastomosis (LVA) involves the surgical creation of an anastomosis between one or more lymphatic vessels and adjacent veins, allowing lymphatic fluid to bypass obstructed pathways and drain into the venous system.^20^^,^^67^ While LVA provides immediate benefits in peripheral lymphedema, its application to neurodegenerative disease remains experimental.^20^ Preliminary reports in small cohorts of AD patients suggest potential cognitive improvement following cervical LVA. Lu et al. reported improvements in Minimum Mental State Examination (MMSE) and Montreal Cognitive Assessment (MoCA) scores in a single patient at 9 months, yet findings were experimental and uncontrolled.^68^ As part of the “Cervical Shunting to Unclog cerebral Lymphatic Systems” (CSULS) trial, Xia et al. reported remarkable cognitive recovery and complete resolution of depressive mood, as well as improvements in brain glucose metabolism in the right frontal lobe 5 weeks after bilateral cervical LVA in a single AD patient.^69^ Furthermore, Chen et al. found the p-tau181/Aβ42-ratio to predict LVA’s effectiveness in a prospective cohort of 26 CE patients.^70^ These findings, however, derive from uncontrolled cohort studies with limited follow-up and must be interpreted cautiously. Additionally, these preliminary reports suggest rapid improvements after cervical LVA, which stands in contrast to the protracted course of AD development typically evolving slowly over decades.^2^^,^^3^ One possibility is that LVAs may preferentially enhance the clearance of still soluble aggregates, thereby alleviating acute neurotoxic inflammation without immediately mobilizing long-standing deposits.^71^ However, whether the already aggregated proteins can be effectively mobilized and eliminated through enhanced lymphatic drainage remains an open mechanistic question and longitudinal studies with biomarker and imaging endpoints will be required to determine if these early clinical observations translate into long-term clinical improvements. Importantly, no data exist on the clinical use of LVA in PD.

Notably, in extremity lymphedema, LVA is typically performed distal to the obstruction.^72^ By analogy, cervical LVA may be physiologically justified in patients with prior neck dissection and subsequent disruption of lymphatic pathways, as the anastomosis provides an alternative pathway into the venous system. In contrast, there is no evidence that LVA augments lymphatic drainage in individuals without proximal obstruction. Notably, most studies employed retroauricular or subauricular approaches, which effectively create a proximal (rather than a distal) shunt, if the brain itself is defined as the obstructed site. This methodological choice may partially account for the lack of statistically significant improvements in research to date. Moving forward, future research should aim to identify target lymphatic vessels directly involved in neurolymphatic drainage and assess long-term biomarker-based outcomes.

### Vascularized lymph node transfer

Vascularized lymph node transfer (VLNT) entails the autologous transplantation of functional lymph nodes from various potential donor sites to a recipient area, where they are anastomosed to the artery and vein. The vascularized lymph node flap then secretes VEGF-C, which promotes lymphangiogenesis.^65^^,^^73^ The thereby established intranodal shunt facilitates lymphatic drainage and fluid clearance through the transplanted lymph nodes into the pedicle vein. In contrast to LVA, VLNT does not require functional patent lymphatic vessels, making it particularly suitable for more advanced stages of extremity lymphedema.^19^^,^^74^ However, its more invasive approach (e.g., donor and recipient site) is associated with a substantially higher risk of postoperative complications.^19^^,^^74^ In an experimental case series of two patients diagnosed with symptomatic communicating hydrocephalus, Wu et al. performed VLNT through a durotomy into the intracranial space.^75^ Through direct injection of indocyanine green (ICG) into the subdural space, absorption was then visualized through the lymph node flap and through the venous pedicle into the venous system, demonstrating the feasibility of intracranial VLNT.^75^ To the best of our knowledge, there are no records of VLNT’s successful use in neurodegenerative diseases, neither in humans nor in animal models. Further research is required to analyze the feasibility and efficacy of both intracranial and cervical VLNT in neurodegenerative diseases.

### Systemic effects of lymphatic reconstruction

Emerging evidence suggests that lymphedema may exhibit characteristics of a systemic condition rather than only being restricted to local fluid accumulation.^76^ This perspective has led to the hypothesis that lymphatic reconstruction could enhance lymphatic drainage not only at the peripheral surgical site, but throughout the entire lymphatic system. Supporting this hypothesis, Yang et al. demonstrated reductions in muscle edema after LVA not only in the affected, lymphedematous leg, but also on the contralateral, “unaffected” limb.^77^

Impaired lymphatic drainage is also associated with chronic inflammation and elevated circulating cytokines.^76^ Most notably, prior research has shown that the systemic immune-inflammation index (SII) and systemic inflammation response index (SIRI), which can be calculated from a regular complete blood count, are also linked to cognitive performance and post-stroke cognitive impairments.^78^^,^^79^ These findings suggest that addressing systemic inflammation by restoring systemic lymphatic function may concurrently reduce neuroinflammation and consequently promote neuroprotection.^71^

### Surgical complications and ethical considerations

It is important to recognize that patients with AD and PD are often older, medically frail, and may have varying degrees of cognitive impairment. These factors substantially heighten perioperative risk. Although surgical enhancement of neurolymphatic clearance is compelling, the risk of intracranial hemorrhage due to pressure gradient backflow remains a major concern, and even minor complications may result in severe or irreversible outcomes.^80^ Any potential neurocognitive benefit must therefore be carefully weighed against these risks in the context of diseases that currently lack curative options. The risk-benefit imbalance also raises the question of whether non-invasive alternatives, such as manual cervical lymphatic drainage, could potentially yield similar effects. Moreover, the ethical justification for performing procedures that are currently classified as highly experimental will require extensive regulatory guidance.

Interpretation of these early clinical experiences must also consider potential sources of bias. When patients are required to cover substantial treatment costs, surgical centers have a financial incentive to emphasize positive outcomes. Furthermore, the introduction of robotic-assisted microsurgical platforms introduces an additional layer of commercial interest. Independent validation in academic settings, free from financial or technological conflicts of interest, will be essential to determine whether neurolymphatic modulation provides true benefit.

## Conclusions and outlook

Recent evidence suggests that impaired lymphatic drainage contributes to the multifactorial pathogenesis of both AD and PD. Consequently, neurolymphatic modulation may offer potential therapeutic strategies for millions of people affected by these currently incurable conditions. However, the standard translational research pathway - from laboratory studies to animal models to human trials—needs to be strengthened in this rapidly emerging field. As outlined above, surgical interventions have been implemented in human patients with still limited preclinical evidence. To advance this, three main research areas must be prioritized:

- Mapping the neurolymphatic system in humans: Further anatomical studies are required to define the structures involved in neurolymphatic clearance. Both ISF and CSF pathways as well as their extracranial connections to the dcLNs must be mapped to aid in the identification of optimal sites for surgical interventions. Such work could enable the identification of target vessels suitable for LVA. Without this knowledge, the procedure remains essentially “blind”, relying on trial-and-error rather than evidence-based anatomical targeting.
- Preclinical neurolymphatic modulation models: Animal models must analyze the feasibility and quantify the effectiveness of both pharmacological (e.g., VEGF-C, Oxytocin, Vitamin D) and surgical interventions (e.g., LVA, VLNT) in promoting neurolymphatic function. Importantly, their effect on cognitive and motor function must be further studied.
- Pilot clinical trials: Once preclinical data have demonstrated therapeutic potential, clinical trials should address the safety and efficacy of neurolymphatic modulation in humans, with long-term follow-up to assess the durability of treatment effects.

Importantly, existing translational efforts have focused almost exclusively on AD, while the potential relevance of neurolymphatic modulation remains largely unexplored, despite a strong pathophysiological rationale. Given the complexity of this emerging field and patient safety as a primary priority, we encourage our colleagues to document, report, and publish outcomes at each stage of investigation in a joint effort to advance neurolymphatic research. In summary, neurolymphatic modulation represents a groundbreaking development in the understanding and possible treatment of neurodegenerative disorders. By encouraging interdisciplinary collaboration between plastic surgery, neurosurgery, psychiatry, dementia specialists, and many more, we may unlock new opportunities to slow disease progression and improve quality of life for patients worldwide.

## Author contributions

The study was conceptualized by all authors. AF performed the primary literature search and wrote the original draft. Project administration and supervision was performed by AF and EAK. All authors read, reviewed and approved the final manuscript.

## Declaration of competing interest

The authors declare that they have no competing interests.
